# Time to diagnose and time to surgery in patients presenting with necrotizing fasciitis: a retrospective analysis

**DOI:** 10.1007/s00068-025-02816-8

**Published:** 2025-03-18

**Authors:** Murad S. Alahmad, Ayman El-Menyar, Husham Abdelrahman, Meiad A. Abdelrahman, Fahad Aurif, Nissar Shaikh, Hassan Al-Thani

**Affiliations:** 1 Trauma Surgery, Hamad Medical Corporation (HMC), Hamad General Hospital (HGH), Doha, Qatar; 2 Clinical Research, Trauma & Vascular Surgery, HGH, PO Box 3050,, Doha, Qatar; 3 Clinical Medicine, Weill Cornell Medicine, Doha, Qatar; 4 Medical Education, HMC, Doha, Qatar; 5 Department of Surgery, General Surgery, HGH, HMC, Doha, Qatar; 6 Surgical Intensive Care Unit, HGH, HMC, Doha, Qatar

**Keywords:** Necrotizing fasciitis, Time to diagnosis, Time to intervention

## Abstract

**Background:**

Necrotizing Fasciitis (NF) is a life-threatening infection characterized by rapid tissue destruction and high mortality. The role of timely diagnosis and surgical intervention in improving patient outcomes remains debated. This study investigates the impact of “time to diagnosis” (TTD) and “time to surgical treatment” (TTS) on the outcomes of NF patients, with a specific focus on the first six hours of critical diagnosis.

**Methods:**

A retrospective analysis was conducted for patients hospitalized with NF between June 2016 and June 2023. Demographic data, comorbidities, clinical features, treatment, and outcomes were analyzed. The study stratified patients based on TTD (early (≤ 6 h) vs. delayed (> 6 h) and TTS (≤ 6 vs. > 6 h). Outcomes included severity scores, intensive care unit admission, length of stay (LOS), and mortality.

**Results:**

One hundred and twenty-one patients were diagnosed with NF with a mortality rate of 10%. Early diagnosis (≤ 6 h) was associated with lower mortality (5.7% vs. 13.2%) and shorter LOS (17 vs. 27 days) compared to delayed diagnosis. Early diagnosis was associated with a lower Sequential Organ Failure Assessment (SOFA) score compared to delayed diagnosis (*p* = 0.02). A combined analysis of TTD and TTS revealed that the group with early diagnosis and early treatment (TTD and TTS were ≤ 6 h) had a 3% mortality rate, and 7% of them had a SOFA score > 9. In contrast, delayed diagnosis (TTD > 6 h) was significantly associated with increased mortality, regardless of the TTS.

**Conclusion:**

Timely diagnosis within 6 h is crucial for improving outcomes in NF. While early surgical intervention is vital, our findings suggest that the time to diagnosis and subsequent resuscitation efforts may significantly impact survival. This study highlights the importance of optimizing early recognition and diagnosis in the emergency room to reduce delays and improve patient prognosis in NF. Further multicenter studies are needed to validate these findings and refine clinical protocols.

**Supplementary Information:**

The online version contains supplementary material available at 10.1007/s00068-025-02816-8.

## Introduction

Necrotizing Fasciitis (NF) is a rare but fatal infection. It is characterized by progressive subcutaneous tissue and fascia destruction with severe systemic inflammatory response. Because of its aggressive character, it is frequently called a “flesh-eating disease” [1]. The etiology of NF is not well understood, but it is often preceded by trivial trauma, such as a superficial burn, insect bite, or even intramuscular injection [2, 3]. NF is not very common; there are 0.3 to 15 instances of NF in every 100,000 people [4].

NF has been recognized as an emergency surgical clinical condition. Despite the evolution of medical treatment, diagnosis is still challenging, and the associated mortality rate remains high at 24–34% [5, 6]. The rapid progression of NF and its subtle early symptoms are significant factors that often delay diagnosis and treatment [4]. It is also frequently misdiagnosed as cellulitis, further delaying appropriate care and contributing to high mortality rates [7].

Several tools, such as the Laboratory Risk Indicator for Necrotizing Fasciitis (LRINEC) score, have been developed to aid in diagnosis and prognosis. Still, no single test can definitively diagnose NF without clinical correlation [8, 9, 10]. A high index of clinical suspicion remains paramount in the diagnosis of NF. Early diagnosis and treatment of NF are crucial to stopping disease progression and preventing severe consequences. Therefore, improving clinical outcomes involves recognizing and managing factors that predict adverse results for patients with NF. One key modifiable factor that affects disease outcomes is the time between a patient’s initial presentation, diagnosis, and surgery [11].

Several studies have reported conflicting findings regarding the impact of time to surgical intervention as an independent predictor of outcomes in NF. Hadeed et al. [12] identified time to surgery as a predictor of prolonged Intensive Care Unit (ICU) and hospital length of stays (LOS). In contrast, prior studies reported that delayed surgery independently predicted mortality [13–14]. However, other studies failed to demonstrate a significant association between time to surgery and outcomes [15, 16], indicating the presence of additional independent predictors of mortality. Among these, the Sequential Organ Failure Assessment (SOFA) score stands out as a reliable mortality predictor, particularly in NF cases involving the perineum, as shown by Azmi et al. [17]. Moreover, age, seasonal variation, and comorbidities such as diabetes mellitus and renal failure have been evaluated as predictors of morbidity and mortality following NF in several studies [6, 18, 19, 20, 21, 22, 23, 24, 25, 26].

There is still a lack of a clear definition of ‘How early should intervention be?’ In the literature, most studies use “time to surgery or time to intervention” as a predictor of morbidity and mortality, and this is a general term to apply. The time spent in the emergency room (ER) before diagnosis is crucial and does impact the clinical outcome. However, the time to intervene needs more elaboration as well. This study aims to explore the impact of early diagnosis and early treatment of NF on the patient’s outcomes.

## Patients and methods

We retrospectively reviewed all NF adult male and female patients who were admitted from June 2016 until June 2023. We analyzed demographic data, medical history (comorbidities), infection distribution, microbiological characteristics, clinical features, hospital course, and outcomes. The diagnosis was based on clinical suspicion and confirmed by intraoperative findings [27]. A combination of clinical evaluation, laboratory tests, and imaging studies is needed for NF diagnosis. It tends to present with a triad of swelling, severe pain, and erythema. Key diagnostic criteria include severe pain out of proportion to physical findings extending beyond the involved skin and rapidly spreading, erythema, skin discoloration and swelling of the affected area, palpable crepitus and paraesthesia over the affected area, blistering or skin necrosis, systemic manifestation [28, 29]. The intraoperative description includes foul “dishwasher” discharge and easily dissected tissue by finger fraction [28, 29]. Histological examination shows necrotizing inflammation with vascular thrombosis. Though not entirely specific, frozen sections can confirm fascial necrosis, vessel thrombosis, and acute inflammation [30].

Patients with intraoperative findings inconsistent with NF diagnosis were excluded. Also, pediatric patients and adults with incomplete information about the timings of presentation, diagnosis, and treatment were excluded. A comparative analysis of demographic, clinical, and laboratory parameters and outcomes in patients with NF was performed. The severity and mortality based on time to diagnosis (TTD) in association with time to surgical treatment (TTS) were also analyzed. In this study, patients were stratified based on TTD and TTS, emphasizing the critical period spent in the ER before surgical intervention. We hypothesized that early TTD and TTS are associated with better outcomes in NF patients. This initial period significantly impacts patients’ outcomes, with a specific focus on the first six hours as a crucial cut-off point for comparison, in alignment with existing literature [12].

### Definitions

TTD is the time for the presentation at the emergency department until the surgical team diagnoses the patient. **TTS** is the time elapsed between diagnosis and surgical intervention (it does not reflect the time from presentation to treatment). The Sequential Organ Failure Assessment (**SOFA**) score is a clinical tool used to assess the extent of a patient’s organ dysfunction and predict the risk of severe sepsis and mortality. For instance, during an intensive care unit stay, the estimated risk of mortality may reach 25% for a maximum SOFA score of 0–9 and 40–60% for a score of 10–14 [31]. It is calculated using parameters such as the ratio of partial pressure arterial oxygen and a fraction of inspired oxygen (PaO2/FiO2), platelets count, bilirubin level, Glasgow coma score, mean arterial pressure, use of vasopressors, creatinine level, and urine output [32, 33]. The **LRINEC** score is a diagnostic scoring tool developed to help differentiate necrotizing fasciitis from other soft tissue infections, such as cellulitis. It uses a set of laboratory values to assess the likelihood of NF as well as the prognosis [10]. LRINEC score ≥ 6 was associated with a risk of sepsis and mortality. At presentation, it was calculated using laboratory results of six variables: C-reactive protein, white blood cell count, hemoglobin, sodium level, creatinine, and glucose.

### Statistical analysis

This study did not require pre-set sample size calculation as we intended to include all the hospitalized cases of NF over a specific period. Data were presented as counts (percentages), mean (standard deviation), or medians (interquartile range) whenever appropriate. Patients were classified and compared based on TTD and TTS. The chi-square test was performed to compare categorical variables in two groups, whereas the Student-t test was used for continuous variables. Mann–Whitney U-test was used for non-parametric data whenever applicable. A two-tailed p-value < 0.05 was considered for statistical significance. Data analysis was carried out using the Statistical Package for Social Sciences version 21 (SPSS Inc., Chicago, IL).

## Results

### Demographics and predisposing factors

One hundred twenty-one (121) cases of NF were included in the study. There were 109 males (90.1%) and 12 females (9.9%) with a mean age of 49.4 ± 12.9 years.

The most common comorbidity associated with NF was DM, found in 75 patients (62%), and hypertension was the second in 43 patients (35.5%). Also, chronic kidney disease and coronary artery disease were found in 25 (20.7%) and 24 (19.8%), respectively. Eleven of the patients (9.1%) had recent surgery in the last two months before illness, and 8 (6.6%) had trauma, either blunt or penetrating. One patient developed NF after an insect bite and one after an Intramuscular injection (0.8%). Table 1 summarizes demographics and the clinical findings. Of the 121 patients reported, 109 (90.1%) survived and 12 (9.9%) died. Sixty-five (65) patients (53.7%) were admitted to the ICU, including all the deceased (12 patients). The median LOS ranged between 14 and 46 days, averaging 26 days.

**Table 1:** Demographic, medical history, infection sites, clinical features and hospital course of patients with necrotizing fasciitis (*N* = 121)

Mean Age	49.4 ± 12.9
Male	109 (90.1)
Shock Index ≥ 1	41 (33.9)
**Medical History (n**,**%)**	
Diabetes Mellitus	75 (62.0)
Coronary Artery Disease	24 (19.8)
Hypertension	43 (35.5)
Chronic Kidney disease	25 (20.7)
Congestive Heart Failure	5 (4.1)
**Recent Medical History (n**,**%)**	
Recent Surgery within 2 months	11 (9.1)
Liver disease	3 (2.5)
Trauma	8 (6.6)
Insect bite	1 (0.8)
History of Intramuscular Injection	1(0.8)
**Distribution of Infection Site (n**, **%)**	
Lower limb/thigh	49 (40.5)
Perineum/Gluteus/Sacral region	59 (48.8)
Abdominal/Groin	12 (9.9)
Upper Limb	7 (5.8)
Neck/Facial	3 (2.5)
**Clinical Features (n**,** %)**	
Pain/Tenderness	115 (95.0)
Swelling	113 (93.4)
Fever	61 (50.4)
**Hospital Course**	
ICU admission (n,%)	65 (53.7)
Hospital LOS (days) (median, IQR)	26 (14–46)
Mortality (n,%)	12 (9.9)
Recurrence (n,%)	6 (5.0)

### Distribution of infection and clinical features

The most common site of infection was the perineal, gluteal, and sacral region in 59 patients (48.8%), followed by the lower limb/thigh in 49 patients (40.5%). Twelve patients (9.9%) had an infection in the abdomen or groin, seven patients (5.8%) had an upper limb infection, and only three cases in the neck or face (2.5%). Pain/tenderness in 115 (95%), local swelling in 113 (93.4%), and fever in 61 (50.4%) were the most common clinical features at presentation.

### Microbiology and cultures

Table 2 summarizes isolated microorganisms. Wound culture was positive in 114 patients (94.2%), while blood culture was positive in only 16 (13.2%). Poly-bacterial infection was isolated in 68 patients (56.2%); the rest were mono-bacterial.

**Table 2:** Microbiological characteristics of necrotizing fasciitis patients (*N* = 121)

Wound culture positive (*n*,%)	114 (94.2)
Blood culture positive	16 (13.2)
Fungus positive	17 (14.1)
Poly-bacterial infection	68 (56.2)
Gram-positive bacteria	84 (69.4)
Gram-negative bacteria	37 (30.6)
Both Gram-positive and Gram-negative	11 (9.1)
Anaerobic bacteria	13 (10.7)
Streptococcus	47 (38.8)
Staphylococcus	31 (25.6)
Escherichia coli	22 (18.2)
Pseudomonas	21 (17.4)
Klebsiella (Kelebsiella)	12 (9.9)
Aeromonas	8 (6.6)
Enterococcus	8 (6.6)
Clostridium	1 (0.8)
Proteus	2 (1.7)
Morganella	4 (3.3)
Prevotella	8 (6.6)

The most common Gram-positive bacteria were group A *Streptococcus and* Staphylococcus in 47 (38.8%) and 31 (25.6%), respectively. *Escherichia coli* was isolated in 22 patients (18.2%), and it was the most common Gram-negative bacteria, followed by Pseudomonas in 21 patients (17.4%). Fungus in the wound was positive in 17 patients (14.1%), and four patients died.

### Comparative analysis of necrotizing fasciitis by time to diagnosis

Analysis of patients with necrotizing is based on the time to diagnosis is demonstrated in Table 3. Patients diagnosed within 6 h were more likely to be male (98.1% vs. 83.8%, *p* = 0.02), present with perineum infections (56.6% vs. 36.8%, *p* = 0.047), and show evident swelling (100.0% vs. 88.2%, *p* = 0.02) compared to those diagnosed after 6 h. Early diagnosis was linked to higher blood glucose levels (15.91 vs. 11.26 mmol/L, *p* = 0.001), while delayed diagnosis was associated with higher creatinine levels (136 vs. 108 µmol/L, *p* = 0.01). Patients diagnosed within 6 h had a median time to treatment of 4 h, while those diagnosed after 6 h had a median time to treatment of 7 h. This difference is statistically significant (*p* = 0.01), indicating that earlier diagnosis is associated with quicker initiation of therapy. No significant differences between groups were found in LRINEC score ≥ 6 (11.3% vs. 7.4%, *p* = 0.70). On the other hand, a larger proportion of patients in the early diagnosis group had lower SOFA scores, with 83.0% scoring below 7, compared to 67.6% in the delayed diagnosis group (*p* = 0.02). Conversely, higher SOFA scores (above 9) were more prevalent in the delayed diagnosis group, with 30.9% of these patients scoring above 9, compared to only 11.3% in the early diagnosis group. Mortality was higher in the delayed group (13.2% vs. 5.7%, *p* = 0.24), though not statistically significant but clinically it is. The length of stay (LOS) was shorter in the early group (17 vs. 27.5 days, *p* = 0.05). ICU admission rates were similar (54.7% vs. 52.9%, *p* = 0.99).

**Table 3:** Comparative analysis of demographic, clinical and laboratory parameters, and outcomes in patients with necrotizing fasciitis by time to diagnosis (TTD)

	TTD < 6 h. (*n* = 53)	TTD ≥ 6 h. (*n* = 68)	*P*-value
Age	47.7 ± 11.5	50.7 ± 13.7	0.199
Male	52 (98.1)	57 (83.8)	0.021
Shock index ≥ 1	20 (37.7)	21 (31.0)	0.356
Diabetes Mellitus	35 (66.0)	40 (58.8)	0.387
Lower limb/thigh infection	16 (30.2)	33 (48.5)	0.064
Perineum infection	30 (56.6)	25 (36.8)	0.047
Pain/Tenderness	52 (98.1)	63 (92.7)	0.341
Swelling	53 (100.0)	60 (88.2)	0.027
Fever	28 (52.8)	33 (48.5)	0.775
White Blood Cell count (mean ± SD)	20.27 ± 8.48	20.75 ± 9.51	0.774
Platelet count (mean ± SD)	304.00 ± 209.36	277.06 ± 129.96	0.392
Hemoglobin (mean ± SD)	11.43 ± 2.25	11.09 ± 2.72	0.466
Sodium level (mean ± SD)	131.77 ± 5.36	132.48 ± 6.67	0.533
C-Reactive Protein (mean ± SD)	290.52 ± 140.00	300.61 ± 129.73	0.69
Glucose (mean ± SD)	15.91 ± 8.96	11.26 ± 6.08	0.001
Creatinine level (median, IQR)	108 (76–144)	136.00 (90.00–305.00)	0.011
Total Bilirubin (median, IQR)	13.00 (10.00–27.00)	17.00 (10.00–30.00)	0.31
Procalcitonin (median, IQR)	3.04 (0.48–8.07)	4.00 (1.02–23.70)	0.264
LRINEC ≥ 6	6 (11.3)	5 (7.4)	0.707
SOFA score			
• < 7	44 (83.0)	46 (67.6)	0.021
• 7–9	3 (5.7)	1 (1.5)	
• > 9	6 (11.3)	21 (30.9)	
Wound culture positive (n,%)	51 (96.2)	63 (92.7)	0.657
Blood culture positive	7 (13.2)	9 (13.2)	0.962
Fungus positive	5 (9.4)	12 (17.7)	0.305
Gram-positive bacteria	38 (71.7)	46 (67.7)	0.779
Gram-negative bacteria	14 (26.4)	23 (33.8)	0.497
Gram-positive and Gram-negative	3 (5.7)	8 (11.7)	0.401
Number of debridement (median, IQR)	2 (1–3)	2 (2–3)	0.968
Septic shock	14 (26.4)	19 (28.0)	0.916
ICU admission (n,%)	29 (54.7)	36 (52.9)	0.992
Hospital LOS (days) (median, IQR)	17.00 (10.00–42.00)	27.50 (16.00–46.00)	0.051
Mortality (n,%)	3 (5.7)	9 (13.2)	0.24
Time to treatment median, IQR	4 (3–7)	7 (4–12)	p 0.01

SOFA: Sequential Organ Failure Assessment; IQR: Interquartile range; LRINEC: Laboratory Risk Indicator for Necrotizing Fasciitis; LOS: Length of stay

### Comparative analysis of necrotizing fasciitis by TTS

Analysis of patients with necrotizing based on the TTS is shown in Table 4. Lower limb infections were more common in the < 6 h group (52.5% vs. 34.9%, *p* = 0.046), whereas perineum infections were more prevalent in the ≥ 6 h group (60.0% vs. 31.2%, *p* = 0.003). The levels of glucose (12.85 ± 7.32 vs. 13.74 ± 8.29, *p* = 0.53) and creatinine (117.00 vs. 116.00, *p* = 0.77) did not significantly differ between early or delayed treatment. Patients treated within 6 h had a significantly shorter time to diagnosis (median of 5 h vs. 8 h, *p* = 0.02). A high LRINEC score (≥ 6) was observed in both groups at comparable rates, 82.0% versus 85.0%, with *p* = 0.69. Patients who received treatment within 6 h had lower SOFA scores overall, with 81.7% of these patients scoring below 7, compared to 67.2% in the delayed treatment group (*p* = 0.04). In contrast, a significantly higher proportion of patients in the delayed treatment group had SOFA scores above 9 (31.1% vs. 13.3%, *p* = 0.04). ICU admission (55.7% vs. 51.7%, *p* = 0.96), LOS (33.00 vs. 21.50 days, *p* = 0.10), and mortality rates (11.5% vs. 8.3%, *p* = 0.99) were comparable between the two groups.

**Table 4:** Comparative analysis of demographic, clinical and laboratory parameters, and outcomes in patients with necrotizing fasciitis by time to treatment (TTS)

	TTS < 6 h. (*n* = 60)	TTS ≥ 6 h. (*n* = 61)	*P*-value
Age	50.2 ± 14.2	48.6 ± 11.3	0.515
Male	52 (85.3)	57 (95.0)	0.136
Shock index ≥ 1	16 (26.2)	17 (28.3)	0.405
Diabetes Mellitus	40 (65.6)	35 (58.3)	0.527
Hypertension	23 (37.1)	20 (33.9)	0.713
cardiovascular disease	15 (24.3)	14 (21.8)	0.892
Chronic Kidney Disease	14 (22.6)	11 (18.6)	0.593
Lower limb/thigh infection	32 (52.5)	21 (34.9)	0.046
Perineum infection	19 (31.2)	36 (60.0)	0.003
Pain/Tenderness	56 (91.8)	59 (98.3)	0.217
Swelling	54 (88.5)	59 (98.3)	0.071
Fever	31 (50.8)	30 (50.0)	0.802
White Blood Cell count (mean ± SD)	20.88 ± 8.89	20.19 ± 9.25	0.676
Platelet count (mean ± SD)	301.45 ± 211.04	276.46 ± 113.88	0.424
Hemoglobin (mean ± SD)	11.06 ± 2.89	11.42 ± 2.09	0.437
Sodium level (mean ± SD)	132.75 ± 6.28	131.56 ± 5.92	0.286
C-Reactive Protein (mean ± SD)	288.57 ± 142.30	303.84 ± 125.52	0.543
Glucose (mean ± SD)	12.85 ± 7.32	13.74 ± 8.29	0.535
Creatinine level (median, IQR)	117 (77–239)	116 (90–190)	0.779
Total Bilirubin (median, IQR)	16.50 (11.00–27.75)	13 (10–29.75)	0.749
Procalcitonin (median, IQR)	2.41 (0.58–15.50)	4.77 (1.02–15.80)	0.352
Time to diagnosis: median, IQR	5 (2–10)	8 (5–12)	0.02
LRINEC ≥ 6	50 (82.0)	51 (85.0)	0.691
SOFA score			
• < 7	49 (81.7)	41 (67.2)	0.04
• 7–9	3 (5.0)	1 (1.6)	
• > 9	8 (13.3)	19 (31.1)	
Wound culture positive (n,%)	57 (93.4)	57 (95.0)	0.995
Blood culture positive	8 (13.1)	8 (13.3)	0.995
Fungus positive	8 (13.1)	9 (15.0)	0.81
Gram-positive bacteria	43 (70.5)	41 (68.3)	0.995
Gram-negative bacteria	21 (34.4)	16 (26.7)	0.274
Gram-positive and Gram-negative	8 (13.1)	3 (5.0)	0.283
Number of debridement (median, IQR)	2.00 (1.00–4.00)	2.00 (2.00–3.00)	0.765
Septic shock	16 (26.2)	17 (28.3)	0.706
ICU admission (n,%)	34 (55.7)	31 (51.7)	0.964
Hospital LOS (days) (median, IQR)	33.00 (16.00–46.00)	21.50 (11.00–42.75)	0.107
Mortality (n,%)	7 (11.5)	5 (8.3)	0.995

SOFA: Sequential Organ Failure Assessment; IQR: Interquartile range; LRINEC: Laboratory Risk Indicator for Necrotizing Fasciitis; LOS: Length of stay

### Severity and mortality based on time to diagnosis in association with TTS

Table 5 highlights the relationship between TTD, TTS, and patient outcomes. The entirely delayed group (TTD > 6 h & TTS > 6 h) had the poorest outcomes, with 39% of patients presenting with a SOFA score > 9, 79% with a LRINEC score > 6, and a hospital mortality rate of 13.2%. The delayed diagnosis with the early treatment group (TTD > 6 h & TTS < 6 h) showed slightly lower severity, with 20% having SOFA > 9 and 70% an LRINEC score > 6, yet had a similar mortality rate of 13.3%. These findings highlight that early treatment alone does not compensate for the impact of delayed diagnosis on mortality.

**Table 5:** Severity of NF and mortality based on time to diagnose in association with time to treat

	TTD < 6 h&TTS < 6 h	TTD < 6 h&TTS > 6 h	TTD > 6 h&TTS < 6 h	TTD > 6 h&TTS > 6 h
**Number of patients**	30 (24.8%)	23 (19%)	30 (24.8%)	38 (31.4%)
**SOFA > 9**	7%	17%	20%	39%
**LRINEK > 6**	90%	70%	70%	79%
**Hospital mortality**	3%	8.7%	13.3%	13.2%

TTD: time to diagnose, TTS: time to surgical treat

In contrast, the group with early diagnosis and treatment (TTD < 6 h & TTS < 6 h) demonstrated SOFA > 9 in 7% and 3% mortality rate. The early diagnosis but delayed treatment group (TTD < 6 h & TTS > 6 h) exhibited intermediate outcomes, with 17% having SOFA > 9 and a mortality rate of 8.7%.

### NF over the years

The incidence of NF fluctuated slightly over the years. Supplementary file 1 shows the frequency and deaths of necrotizing fasciitis by year (2016–2022). In 2016, 11 NF cases were reported with 2 fatalities, while in 2018, 27 NF cases were reported with no mortality.

## Discussion

NF is a surgical emergency with high morbidity and mortality rates that pose significant challenges in diagnosis and treatment. This study presents the clinical, laboratory, and microbiological characteristics and outcomes of 121 patients diagnosed and hospitalized with NF over six years in a rapidly developing Middle Eastern country. The study’s outcome was based on the time to diagnosis and the time to surgical intervention.

In existing medical literature, it has been noted that NF can develop at any age, although it is most reported in individuals between the ages of 32 and 57, with the average age observed in the current study being 49 years [34, 35]. The areas of the body most frequently affected by NF include the extremities, perineum, head & neck, and truncal regions, with the perineum and lower limbs being the most infected sites in the current study [36].

Symptoms typically associated with NF, such as pain, tender local swelling, and fever, were commonly observed in the current group, consistent with earlier reports [19, 37]. Moreover, the frequent co-morbidities related to NF encompass diabetes mellitus, malignancy, chronic cardiac disease, peripheral vascular disease, chronic renal disease, and immune suppression [7]. Notably, among these co-morbidities, diabetes mellitus, hypertension, and renal impairment were the most encountered in the current study group.

Previous studies on NF have addressed a variety of potential predictive factors. El-Menyar et al. [10] included 294 NF patients and focused on the utility of LRINEC scores, dividing patients into those with scores < 6 and ≥ 6. Age and LRINEC scores were significantly associated with mortality and septic shock in univariable analyses. However, the time from presentation and diagnosis to surgery is the most important modifiable element in the outcomes of patients with NF [11, 12]. Most studies focused on the impact of time to surgery or time to intervention on the outcome of NF patients. However, published reports remain inconsistent about which time cut-off for surgical intervention is a significant independent predictor. Two meta-analyses by Gelbard et al. and Nawjin et al. [38, 39] found that time to surgery was associated with decreased mortality “if” surgery was done within 12 h of presentation. Nawjin et al. described the 12-hour golden period as the “minimal” golden period for intervention but recommended an earlier 6-hour period. Our study and others [12, 15, 16, 40] have failed to find time for surgery to be significant in multivariable modeling to define the predictors of mortality. We believe that the time spent in the emergency room before diagnosis (TTD) is crucial, impacts the outcome, and goes side by side with time to treatment. Initially, we studied the impact of early versus late diagnosis and early versus late treatment on the outcomes of NF, including ICU admission, mortality, and length of stay (LOS). We found that early diagnosis (within 6 h) was associated with better outcomes, including lower mortality rates (3% vs. 13.2%) and shorter LOS (17 days vs. 27 days), compared to delayed diagnosis. In terms of time to treatment, for those who received treatment within 6 h, the outcomes were worse, which suggests that early diagnosis may play a more critical role in improving outcomes than early treatment alone. When examining the combined effect of TTD and TTS, we found that the group with both early diagnosis and early treatment (TTD < 6 h & TTS < 6 h) had the best outcomes, with only 7% presenting with a SOFA score > 9 and a 3% mortality rate. In contrast, among groups with delayed diagnosis (TTD > 6 h), mortality rates were similar regardless of whether treatment was early or late, with 13.3% in the early treatment group (TTD > 6 h & TTS < 6 h) and 13.2% in the late treatment group (TTD > 6 h & TTS > 6 h). These findings suggest that delayed diagnosis significantly impacts mortality outcomes. Notably, the SOFA score, an established predictor of mortality in NF [6, 18], demonstrated a clear relationship with delays in diagnosis and treatment. The percentage of patients with SOFA scores > 9 increased progressively across groups, ranging from 7% in the early diagnosis and treatment group to 39% in the group with both delayed diagnosis and treatment (TTD > 6 h & TTS > 6 h). This underscores the critical impact of delayed diagnosis on the severity of organ dysfunction, which directly correlates with increased mortality. Thus, the critical factors may be the time to surgery, the time to diagnosis, and the time to decide to perform surgery. We also believe that better outcomes observed in patients with early diagnosis may be attributed to timely identification and NF-directed resuscitation, including appropriate antibiotics and aggressive fluid management. A study by Tsai et al. [41]. emphasizes the critical role of early and appropriate antibiotic therapy in improving patient outcomes. While it may seem common sense that targeting early antimicrobial administration may also impact the outcome, we couldn’t show this in our study as it needs more precise information about the type of antibiotics used and the timing, and this is one of the limitations.

The period spent in the ER before the patient is labeled as NF is the golden period with a cut-off of 6 h. This constituted the effect of resuscitation done in the ER before surgery. Sometimes, stabilizing the hemodynamic status of a patient with severe NF preoperatively takes more time, and thus, it delays the on-time definitive treatment and negatively impacts the patient’s outcome [42]. In the current study, earlier diagnosis showed better outcomes in terms of mortality (5.7% vs. 13.2%) and LOS (17 vs. 27 days) in the earlier group. Thus, time to diagnosis is of the essence.

The limitations of the present study included the retrospective design, small sample size, single-center data, and lack of reasons for diagnosis delay. However, several issues frequently impede the clinical application of the intended early diagnosis and debridement. Patient delay is a problem that medical personnel cannot easily influence. Many clinical, financial, and social factors affect how long a patient waits to seek medical attention [43]. So, delay in presentation may also have an impact, which we didn’t include in our study, and this is another limitation. Next, hospital delays and poor experiences with NF due to its low incidence may delay or even misdiagnose NF with other soft tissue infections. Moreover, the improved outcomes in patients with early diagnosis can be attributed to timely surgical intervention and the implementation of NF-directed resuscitation measures, including appropriate antimicrobial therapy and aggressive fluid management. These findings stress the importance of optimizing emergency room protocols to prioritize early recognition and stabilization of patients suspected of having NF.

Though we could not elaborate on the impact of time to surgery on clinical outcomes, we believe this should continue to be examined in future studies with multicenter collaboration aiming for a bigger sample and power to explore the impact of time on the clinical outcome of this important and serious disease. Therefore, enhancing diagnostic protocols and training to achieve earlier diagnosis should be a primary focus in the clinical management of this life-threatening condition. Further research is needed to explore strategies for reducing diagnostic delays and to confirm these findings in larger patient cohorts. Although its retrospective design and single-center scope limit this study, it introduces the concept of “time to diagnosis” as an independent predictor of outcomes, which warrants further exploration. The study may have inadequate power in post hoc analysis because of the limited sample size. Therefore, the findings need further investigation in a larger population. This will also allow for conducting robust multivariate logistic regression analysis to assess the independent predictors of the study outcomes. Future research with larger, multicenter cohorts is essential to validate these findings, refine diagnostic protocols, and develop strategies to minimize delays in both diagnosis and treatment. By addressing these challenges, we can improve survival rates and reduce the morbidity associated with necrotizing fasciitis. Lastly, this study has gender-biased findings as most cases are males. However, previous work from the same group reported a higher percentage of males with NF in Qatar with a male-to-female ratio of 3:1 [5]. The influx of male laborers (80% of the population in Qatar are expatriates with a South Asian predominance) has skewed the gender balance, and women represent just one-quarter of the population.

## Conclusion

This study highlights the critical importance of timely diagnosis in improving outcomes for patients with necrotizing fasciitis. Early diagnosis, defined as within six hours of presentation, was associated with significantly lower mortality rates and shorter hospital stay, emphasizing its pivotal role in managing this life-threatening condition. While early surgical intervention is often emphasized, our findings suggest that the time to diagnosis and subsequent resuscitation efforts may have a greater impact on patient outcomes. The strong correlation between delayed diagnosis and higher SOFA scores further underscores the role of early and accurate diagnosis in mitigating disease severity and reducing mortality.

## Electronic supplementary material

Below is the link to the electronic supplementary material.

Supplementary Material 1

## Data Availability

All data are presented in the manuscript, further access needs approval from the medical research center at Hamad medical corporation, after reasonable research request and signed data share agreement.
