# Supplementary material for: Time to diagnose and time to surgery in patients presenting with necrotizing fasciitis: a retrospective analysis
Source: Eur J Trauma Emerg Surg. 2025 Mar 18;51(1):140. doi: 10.1007/s00068-025-02816-8 (PMC11920322; doi:10.1007/s00068-025-02816-8)
Supplement: Supplementary file 1 — Supplementary Material 1 [file 68_2025_2816_MOESM1_ESM.docx]

Supplementary file 1: Frequency and deaths of necrotizing fasciitis by year in Hamad Medical Corporation, Qatar (2016-2022)
